# Supplementary material for: Meta-analytic evidence that sexual selection improves population fitness
Source: Nat Commun. 2019 May 1;10:2017. doi: 10.1038/s41467-019-10074-7 (PMC6494874; doi:10.1038/s41467-019-10074-7)
Supplement: Supplementary file 1 — Supplementary Information [file 41467_2019_10074_MOESM1_ESM.pdf]

Supplementary Information

Meta-analytic evidence that sexual selection improves population fitness

Justin G. Cally, Devi Stuart-Fox and Luke Holman

Contents

Supplementary Methods . . . 2
Literature Search . . . 2
Additions to the Literature Search . . . 3
Inclusion/Exclusion criteria . . . 3
Data Extraction and Effect Size Calculation . . . 3
Supplementary Table 1 . . . 4
Supplementary Table 2 . . . 5
Supplementary Table 3 . . . 7
Supplementary Table 4 . . . 8
Supplementary Table 5 . . . 9
Supplementary Table 6 . . . 10
Supplementary Table 7 . . . 11
Supplementary Figure 1 . . . 12
Supplementary Table 8 . . . 13
Supplementary Table 9 . . . 14
Supplementary Figure 2 . . . 15
Supplementary Table 10 . . . 16
Supplementary Table 11 . . . 17
Supplementary Table 12 . . . 17
Supplementary Table 13 . . . 18
Supplementary Table 14 . . . 18
Supplementary Figure 3 . . . 19
Supplementary Table 15 . . . 19
Supplementary Figure 4 . . . 20
Supplementary Table 16 . . . 20
Supplementary Table 17 . . . 21
Supplementary Table 18 . . . 21
Supplementary Table 19 . . . 22
Supplementary Table 20 . . . 22
Supplementary Figure 5 . . . 23
Supplementary Figure 6 . . . 24
Supplementary Table 21 . . . 24
Supplementary Figure 7 . . . 25
Supplementary Figure 8 . . . 26
Supplementary Figure 9 . . . 27
Supplementary References . . . 28

Supplementary Methods

Our aim was to investigate the effects of sexual selection on population fitness by conducting a meta-analysis on studies that measured fitness related outcomes after experimentally evolving a population under varying levels of opportunity for sexual selection. Here we describe the process of the literature search, data extraction, effect size calculation, formulation of multilevel models and assessing publication bias. We used the PRISMA (Preferred Reporting Items for Systematic Reviews and Meta-Analyses) as a guide during this meta-analysis. The repository used to formulate this document can be found here: https://github.com/JustinCally/SexualSelection. The more extensive HTML version of Supplementary Information is hosted through github pages here: https://justincally.github.io/SexualSelection/

Literature Search

The literature search was conducted under the following conditions:

1. We searched ISI Web of Science and Scopus on 9th June 2017. The two search engines produded a somewhat different set of papers (**see PRISMA Figure in manuscript**).
2. Studies were restricted to those from peer-reviewed and in the English language.
3. We devised a search strategy that sought to find studies which manipulated the presence or strength of sexual selection using experimental evolution, and then measured some proxy of population fitness. As such the search terms were as follows:

ISI Web of Science

We used the following search on ISI Web of Science:

Topic (TS) = "Sexual Selection" OR Promisc* OR Monogam* OR Polygam* OR Polyandr* OR Polygyn* OR "Mate choice"

AND

Topic (TS) = Fitness OR "Population Fitness" OR Deleterious OR "Male Strength" OR Fecund* OR Viability OR Productiv* OR "Reproductive Success" OR "Reproductive Rate" OR Surviv* OR | "Development Rate" OR Extinct* OR "Competitive Success" OR Mortality OR Mass OR "Body Size" OR "Wing Size" OR Emergence OR Mating Rate OR "Mating Propensity" OR Adapt* OR "Novel | Environment" OR "Sexual Conflict" OR "Sexual Antagonis*"

AND

Topic (TS) = Generations OR "Experimental evolution" OR "mutation load"

AND

Research Area (SU) = "Evolutionary Biology"

Scopus

We used the following search on Scopus:

TITLE-ABS-KEY = "Sexual Selection" OR Promisc* OR Monogam* OR Polygam* OR Polyandr* OR Polygyn* OR "Mate choice"

AND

TITLE-ABS-KEY = Fitness OR "Population Fitness" OR Deleterious OR "Male Strength" OR Fecund* OR Viability OR Productiv* OR "Reproductive Success" OR "Reproductive Rate" OR Surviv* | OR "Development Rate" OR Extinct* OR "Competitive Success" OR Mortality OR Mass OR "Body Size" OR "Wing Size" OR Emergence OR Mating Rate OR "Mating Propensity" OR Adapt* OR | "Novel Environment" OR "Sexual Conflict" OR "Sexual Antagonis*"

AND

TITLE-ABS-KEY = Generations OR “Experimental evolution” OR “mutation load”

Additions to the Literature Search

In addition to studies found from the literature search we also included three relevant studies that we found, which were not picked up in the subsequent formal searches[1–3] **see PRISMA Figure in manuscript**.

Inclusion/Exclusion criteria

After removing duplicates papers recovered from both *Web of Science* and *Scopus*, we read the titles and abstracts of the remaining 1015 papers, and removed papers that were not relevant (typically because they were not an empirical study using experimental evolution). This left 130 papers, for which we read the full text and applied the following selection criteria:

- **(1: Study Design)** The study was an experimental evolution study lasting >1 generation
- **(2: Population)** a) The study was conducted using an animal species that was b) dioecious
- **(3: Intervention and Control)** The study experimentally manipulated the strength of sexual selection for at least one generation (e.g. via enforced monogamy or an altered sex ratio)
- **(4: Outcomes)** The study measured a trait that we judged to be a potential correlate of population fitness.

Criterion 4 is somewhat subjective, because there is rarely enough data justify the assumption that a particular trait is (or is not) correlated with population fitness. We therefore relied on our best judgement when deciding which studies to exclude (see **Supplementary Table 1**). The inclusion/exlusion critera as applied to each study are detailed in **Supplementary Table 2**.

Data Extraction and Effect Size Calculation

The rules utilised during the data extraction and effect size calculation were as follows:

1. Arithmatic means, standard deviations/errors and sample sizes were extracted from a paper, supplementary material or a linked data repository (e.g. Data Dryad). This was possible when means and SD were reported in text or in a table. We would preferentially extract data for each experimental evolution line/replicat/family if possible and only extract data for the final reported generation (which was noted down).

2. If we could not find the means and SD in text format we used web-plot digitizer (v.3.12) to extract data from graphs.

3. If means were not reported then we extracted a summary statistic or proportion value, which we could later convert to Hedges g’ using the *compute.es* package[4]. Summary statistics included F, z, t and chi^2. These conversions still required providing sample sizes for each treatment so these needed to be extractable from the study. Some summary statistics were obtained from generalized linear model summary tabels, others from straight forward ANOVAs and then some from more complex analysis such as proportional hazards statistical tests.

4. We also collected various covariates for some of the studies (**Supplementary Table 3**), which are discussed later.

Supplementary Table 1

We classified each of the twenty fitness outcomes into three broad groups – direct, indirect and ambiguous – based on the established link with population fitness, the directionality of the measure. Here we detailed how these outcomes were measured in the primary studies, which are cited in the "Citation" column.

Outcome	Classification	Explanation	Citation
Behavioural Plasticity	Ambiguous	Female kicking against male harassment in different sociosexual contexts for the beetle Callosobruchus maculatus.	[5]
Body Size	Ambiguous	Body size was often recorded to correct for other morphometric traits (e.g. body condition, strength or testes weight). It was measured as either length or dry mass.	[6,7]
Development Rate	Ambiguous	Egg-to adult development time was recorded in several studies and often alongside traits other life-history traits suspected to impact fitness.	[8–10]
Early Fecundity	Ambiguous	Early fecundity was measured (alongside lifetime fecundity) as a life-history trait that may impact lifetime reproductive success. It was defined as either the total or proportional reproductive output in earlier stages of maturity (e.g. within the first 7 days).	[11,12]
Immunity	Ambiguous	Phenoloxidase (PO) activity or parasite load.	[10,13–15]
Male Attractiveness	Ambiguous	Inferred from female preference tests in mice and male ornament size (coloration) in guppies.	[16–18]
Male Reproductive Success	Ambiguous	Measured as the total progeny sired in males.	[9]
Mating Duration	Ambiguous	Mating duration may have variable fitness impacts based on the soiciosexual conditions and extent of sexual conflict. It may be beneficial to have longer mating bouts for a male in a competitive environment however it may be damaging for a female under benign conditions.	[5,19–21]
Pesticide Resistance	Ambiguous	Pesticide resistance was measured both in the presence and absence of pesticides for the insect Tribolium castaneum, it was a binary measure of resistance to knockdown that was incorporated into generalized linear mixed models.	[22]
Mutant Frequency	Indirect	Allele and mutant frequency measured at the population level.	[23,24]
Body Condition	Indirect	Mean body weight of Onthophagus Taurus adjusted for body size (thorax width).	[6]
Fitness Senescence	Indirect	Rate of decline in survival probability across lifespan.	[9,25]
Lifespan	Indirect	Longevity or survival across the entire lifespan or from a given point once under stressful conditions, such as starvation or after females mated in different operational sex ratios.	[26,27]
Mating Frequency	Indirect	Number of mounts by males on females in Tribolium castaneum and Drosophila melanogaster.	[13,20]
Mating Latency	Indirect	Time taken for a male to undertake their first copulatory mount from the time of being first put together with female/s.	[5,9,19–21]
Mating Success	Indirect	Male mating success measured males ability to successfully mate with females. Often in the presence of other males. Mating success of a male against a rival male can be determined via competing a focal male against an irradiated (infertile) competitor, the resulting proportion of eggs hatching are then determined to be a measure of the focal males success. Mating success also included measurements of mating capacity where males were continually presented with females until exhausted, the number of sequential matings were then recorded and mating offence and defence ability. The mating offence and defence capability was estimated via paternity share of a male when in the first mating position (P1) or the second (P2).	[12,21,28–30]
Strength	Indirect	Male pulling strength in the dung beetle, Onthophagus Taurus, measured by attaching weights and measuring the weight the beetle was able to pull.	[7]
Ejaculate Quality and Production	Indirect	Sperm quality and production grouped multiple measured outcomes together, both within a study (28) and during the meta-analysis. This includes sperm size, plug size, testes size, soporific effect, ejaculate weight, accessory gland size, motility, path velocity, sperm longevity.	[5,31–34]
Extinction Rate	Direct	Extinction rate was measured at the population level, either via recording the proportion of extinct lines after a given number of generations or via analysis of extinction rate over consecutive generations via the Weibull baseline hazard distribution.	[35–37]
Offspring Viability	Direct	Offspring viability, also recorded as egg-to-adult viability or embryonic viability, was measured as survival to a certain age (e.g. 1 year or life stage and hatching).	[18,38]
Female Reproductive Success	Direct	A measure of the number of offspring produced by an individual female. Reproductive success was also described as fecundity, number of offspring produced, fertility in females and proportion.	[19,39,40]
Both Reproductive Success	Direct	Similar to Female Reproductive Success, however measurements of offspring produced are sourced from a focal male-female pair, of which either male or female may limit the number of offspring produced.	[37]

Supplementary Table 2

A study was deemed eligible for inclusion in the meta-analysis if it met all four criteria discussed above (referred to by their numbers, 1-4, in this table). We went through these four criteria in a step-wise fashion, for each of the 130 studies for which we read the full text, and noted the first criterion that was failed (if any). For pdf formatting this table has been resized and does not have the paper title and notes. Please refer to the html table on github for the extensive table: https://justincally.github.io/SexualSelection

AuthorYear	Study Design	Population	Intervention and Control	Outcomes	Included	Exclusion Reason
Aguirre (2012)[41]	No				No	1
Ahuja (2008)[42]	No				No	1
Almbro (2014)[7]	Yes	Yes	Yes	Yes	Yes	
Amitin (2007)[43]	Yes	Yes	No		No	3
Antolin (2003)[44]	No				No	1
Arbuthnott (2014)[45]	Yes	Yes	No		No	3
Arbuthnott (2012)[23]	Yes	Yes	Yes	Yes	Yes	
Arbuthnott (2014)[46]	Yes	Yes	No		No	3
Archer (2015)[25]	Yes		Yes	Yes	Yes	
Artieri (2008)[47]	No				No	1
Bacigalupe (2007)[48]	Yes	Yes	Yes	No	No	4
Bacigalupe (2008)[49]	Yes	Yes	Yes	Yes	No	Data not suitable
Barbosa (2012)[50]	No				No	1
Bernasconi (2001)[40]	Yes	Yes	Yes	Yes	Yes	
Bielak (2014)[51]	No				No	1
Blows (2002)[52]	Yes	Yes	Yes	No	No	4
Brommer (2012)[53]	Yes	Yes	Yes	Yes	Yes	
Castillo (2015)[54]	Yes	Yes	No		No	3
Cayetano (2011)[55]	Yes	Yes	Yes	No	No	4
Chandler (2013)[56]	Yes	No			No	2a
Chenoweth (2015)[57]	Yes	Yes	Yes	No	No	4
Chenoweth (2007)[58]	No				No	1
Chenoweth (2008)[59]	Yes	Yes	Yes	No	No	4
Chenoweth (2010)[60]	Yes	Yes	Yes	No	No	4
Crudgington (2005)[61]	Yes	Yes	Yes	Yes	Yes	
Crudgington (2009)[30]	Yes	Yes	Yes	Yes	Yes	
Crudgington (2010)[11]	Yes	Yes	Yes	Yes	Yes	
Debelle (2016)[28]	Yes	Yes	Yes	Yes	Yes	
Demont (2014)[62]	Yes	Yes	Yes	Yes	Yes	
Edward (2010)[19]	Yes	Yes	Yes	Yes	Yes	
Fava (1975)[63]	Yes	Yes	No		No	3
Firman (2011)[39]	Yes	Yes	Yes	Yes	Yes	
Firman (2014)[16]	Yes	Yes	Yes	Yes	Yes	
Firman (2011)[64]	Yes	Yes	Yes	Yes	Yes	
Firman (2015)[65]	Yes	Yes	Yes	Yes	Yes	
Firman (2014)[66]	Yes	Yes	Yes	Yes	Yes	
Firman (2010)[31]	Yes	Yes	Yes	Yes	Yes	
Firman (2011)[67]	Yes	Yes	Yes	Yes	Yes	
Firman (2012)[68]	Yes	Yes	Yes	Yes	Yes	
Fricke (2010)[69]	Yes	Yes	Yes	No	No	4
Fricke (2007)[8]	Yes	Yes	Yes	Yes	Yes	
Fritzsche (2016)[70]	Yes	Yes	Yes	Yes	Yes	
Fritzsche (2014)[32]	Yes	Yes	Yes	Yes	Yes	
Garcia (2015)[71]	Yes	Yes	Yes	Yes	No	Data not suitable
Gay (2009)[72]	Yes	Yes	No		No	3
Gay (2011)[73]	Yes	Yes	No		No	3
Gay (2009)[33]	Yes	Yes	Yes	Yes	Yes	
Grazer (2014)[74]	Yes	Yes	Yes	Yes	Yes	
Grieshop (2016)[75]	Yes	Yes	No		No	3
Hall (2009)[76]	Yes	Yes	No		No	3
Hangartner (2015)[13]	Yes	Yes	Yes	Yes	Yes	
Hangartner (2013)[14]	Yes	Yes	Yes	Yes	Yes	
Hicks (2004)[77]	Yes	Yes	No		No	3
Holland (2002)[78]	Yes	Yes	Yes	Yes	Yes	
Holland (1999)[79]	Yes	Yes	Yes	Yes	Yes	
Hollis (2009)[24]	Yes	Yes	Yes	Yes	Yes	
Hollis (2011)[80]	Yes	Yes	Yes	Yes	Yes	
Hollis (2016)[81]	Yes	Yes	Yes	No	No	4
Hollis (2014)[82]	Yes	Yes	Yes	No	No	4
Hollis (2014)[9]	Yes	Yes	Yes	Yes	Yes	

AuthorYear	Study Design	Population	Intervention and Control	Outcomes	Included	Exclusion Reason
Hollis (2017)[83]	Yes	Yes	Yes	Yes	Yes	
Hosken (2009)[84]	Yes	Yes	Yes	No	No	4
House (2013)[85]	Yes	Yes	Yes	No	No	4
Hunt (2012)[86]	Yes	Yes	Yes	No	No	4
Immonen (2014)[87]	Yes	Yes	Yes	Yes	Yes	
Innocenti (2014)[88]	Yes	Yes	Yes	Yes	Yes	
Jacomb (2016)[22]	Yes	Yes	Yes	Yes	Yes	
Janicke (2016)[89]	Yes	No			No	2b
Jarzebowska (2010)[35]	Yes	Yes	Yes	Yes	Yes	
Klemme (2013)[90]	Yes	Yes	Yes	Yes	Yes	
Long (2012)[91]	Yes	Yes	No		No	3
Lumley (2015)[37]	Yes	Yes	Yes	Yes	Yes	
MacLellan (2012)[92]	No				No	1
MacLellan (2009)[93]	No				No	1
Maklakov (2009)[94]	Yes	Yes	Yes	Yes	Yes	
Maklakov (2009)[95]	Yes	Yes	Yes	No	No	4
Maklakov (2007)[96]	Yes	Yes	Yes	No	No	4
Mallet (2011)[97]	Yes	Yes	No		No	3
Mallet (2011)[98]	No				No	1
Martin (2003)[27]	Yes	Yes	Yes	Yes	Yes	
Martin (2004)[99]	Yes	Yes	Yes	Yes	Yes	
Matsuyama (2009)[100]	Yes	Yes	No		No	3
McGuigan (2011)[29]	Yes	Yes	Yes	Yes	Yes	
McKean (2008)[10]	Yes	Yes	Yes	Yes	Yes	
McLain (1992)[101]	No				No	1
McNamara (2016)[34]	Yes	Yes	Yes	Yes	Yes	
McNamara (2014)[15]	Yes	Yes	Yes	Yes	Yes	
Meffert (2006)[102]	Yes	Yes	No		No	3
Michalczyk (2011)[103]	Yes	Yes	No		No	3
Michalczyk (2011)[20]	Yes	Yes	Yes	Yes	Yes	
Morrow (2008)[104]	Yes	Yes	No		No	3
Nandy (2013)[21]	Yes	Yes	Yes	Yes	Yes	
Nandy (2014)[105]	Yes	Yes	No	Yes	Yes	
Nelson (2013)[17]	Yes	Yes	Yes	Yes	Yes	
Nie (2016)[106]	No				No	1
Palopoli (2015)[107]	Yes	No			No	2b
Partridge (1980)[108]	Yes	Yes	Yes	Yes	Yes	
Pelabon (2014)[18]	Yes	Yes	Yes	Yes	Yes	
Perry (2016)[109]	Yes	Yes	No		No	3
Pischedda (2005)[110]	No				No	1
Pischedda (2006)[111]	No				No	1
Pitnick (2001)[112]	Yes	Yes	Yes	Yes	Yes	
Pitnick (2001)[113]	Yes	Yes	Yes	Yes	Yes	
Plesnar (2011)[38]	Yes	Yes	Yes	Yes	Yes	
Plesnar (2013)[114]	Yes	Yes	Yes	No	No	4
Plesnar (2012)[36]	Yes	Yes	Yes	Yes	Yes	
Power (2014)[115]	Yes	Yes	Yes	Yes	Yes	
Power (2015)[116]	Yes	Yes	Yes	Yes	Yes	
Price (2010)[117]	Yes	Yes	No		No	3
Prokop (2017)[118]	No				No	1
Promislow (1998)[119]	Yes	Yes	Yes	Yes	Yes	
Radwan (2004)[120]	Yes	Yes	Yes	Yes	Yes	
Radwan (2004)[121]	Yes	Yes	Yes	Yes	Yes	
Rundle (2006)[122]	Yes	Yes	Yes	Yes	Yes	
Rundle (2009)[123]	Yes	Yes	Yes	No	No	4
Rundle (2007)[124]	Yes	Yes	No		No	3
Savic (2013)[125]	Yes	Yes	Yes	Yes	No	Data not suitable
Seslija (2008)[126]	Yes	Yes	No		No	3
Sharma (2012)[127]	Yes	Yes	Yes	No	No	4
Sharp (2008)[128]	No				No	1
Sharp (2009)[129]	No				No	1
Simmons (2014)[130]	Yes	Yes	Yes	No	No	4
Simmons (2008)[6]	Yes	Yes	Yes	Yes	Yes	
Simmons (2011)[131]	Yes	Yes	Yes	No	No	4
Simmons (2009)[132]	Yes	Yes	Yes	No	No	4
Snook (2013)[133]	Yes	Yes	Yes	No	No	4
Tilszer (2006)[12]	Yes	Yes	Yes	Yes	Yes	
van (2014)[5]	Yes	Yes	Yes	Yes	Yes	
Whitlock (2000)[134]	Yes	Yes	No		No	3
Wigby (2004)[26]	Yes	Yes	Yes	Yes	Yes	

Supplementary Table 3

Table of effect sizes included in our meta-analysis.

	n
Effect sizes (Total)	459
Publications	65
Blind experiments	54
Effect sizes (Enforced monogamy)	241
Effect sizes (Ambiguous)	144
Effect sizes (Indirect)	141
Effect sizes (Direct)	174
Effect sizes (Stressful)	92
Effect sizes (Benign)	337
Effect sizes (Male)	189
Effect sizes (Female)	219
Effect sizes (Both sexes)	51
Different species	15
Effect sizes (Beetle)	116
Effect sizes (Fly)	254
Effect sizes (Mouse)	40
Effect sizes (Nematode)	9
Effect sizes (Mite)	25
Effect sizes (Cricket)	6
Effect sizes (Guppy)	9

Supplementary Table 4

Table of fitness outcomes included in our meta-analysis by sex.

Parameters	Both	Female	Male	Total
Behavioural Plasticity	0	2	0	2
Body Condition	0	0	1	1
Body Size	2	13	11	26
Both Reproductive Success	12	0	0	12
Development Rate	5	1	1	7
Early Fecundity	0	14	0	14
Ejaculate Quality and Production	0	0	23	23
Extinction Rate	4	0	0	4
Female Reproductive Success	0	102	0	102
Fitness Senescence	0	3	3	6
Immunity	5	15	15	35
Lifespan	0	35	3	38
Male Attractiveness	0	0	6	6
Male Reproductive Success	0	0	42	42
Mating Duration	0	1	9	10
Mating Frequency	0	6	5	11
Mating Latency	0	1	12	13
Mating Success	0	0	39	39
Mutant Frequency	6	0	2	8
Offspring Viability	15	26	15	56
Pesticide Resistance	2	0	0	2
Strength	0	0	2	2

Supplementary Table 5

These estimates are presented in the text of the Results section. The test statistic is either the p-value (REML) or Bayes factor (BF) comparing the effect size to zero (Bayesian).

Method	Grand__mean__effect__size__g	Lower__95__CI	Upper__95__CI	Test__statistic
REML	0.24	0.055	0.43	0.011
Bayesian	0.25	-0.0074	0.51	35

Supplementary Table 6

The predicted effect size for each of the three fitness trait classes (Ambiguous, Indirect and Direct) that are presented in Figure 1 in the manuscript. This table presents both Bayesian and REML predictions with some discrepencies in the estimated error margins. Figure 1 within the manuscript uses REML predictions.

Relationship to Fitness	Bayes Prediction	Bayes SE	Bayes LCI	Bayes UCI	n	BF	REML Prediction	REML SE	REML LCI	REML UCI
Indirect	0.24	0.098	0.0326	0.43	141	59	0.24	0.057	0.132	0.36
Ambiguous	0.20	0.098	-0.0016	0.39	144	38	0.21	0.058	0.093	0.32
Direct	0.13	0.097	-0.0790	0.31	174	11	0.13	0.057	0.019	0.24

Supplementary Table 7

Summary of model predictions for 22 fitness components. In Supplementary Figure 1 these values are presented as a text overlay using the Bayesian values. Additionally, Bayes Factors (BF) are presented as the likelihood ratio that the effect size is greater than 0. Where values greater than 1 correspond to higher likelihood of the effect size being positive and values less than 1 suggest that the effect size is more likely to be negative. The right side of the table provides the REML estimates with SE and 95

Fitness Component	Bayes Prediction	Bayes SE	Bayes LCI	Bayes UCI	n	BF	REML Prediction	REML SE	REML LCI	REML UCI
Behavioural Plasticity	0.282	0.19	-0.090	0.66	2	1.5e+01	0.279	0.172	-0.057	0.616
Body Size	0.380	0.11	0.159	0.62	26	2.2e+02	0.378	0.078	0.225	0.532
Development Rate	0.517	0.15	0.223	0.82	7	6.7e+02	0.515	0.125	0.270	0.761
Early Fecundity	0.281	0.16	-0.027	0.59	14	2.7e+01	0.280	0.134	0.017	0.543
Immunity	-0.422	0.14	-0.702	-0.15	35	2.6e-03	-0.419	0.111	-0.636	-0.201
Male Attractiveness	0.302	0.14	0.031	0.59	6	5.8e+01	0.298	0.111	0.081	0.515
Male Reproductive Success	0.155	0.12	-0.072	0.39	42	1.4e+01	0.152	0.080	-0.005	0.310
Mating Duration	0.422	0.12	0.186	0.67	10	3.1e+02	0.420	0.089	0.247	0.594
Pesticide Resistance	1.051	0.49	0.095	2.01	2	6.4e+01	1.076	0.457	0.180	1.973
Mutant Frequency	0.319	0.36	-0.387	1.02	8	4.5e+00	0.294	0.334	-0.361	0.949
Body Condition	-1.235	0.32	-1.871	-0.63	1	0.0e+00	-1.227	0.305	-1.825	-0.629
Fitness Senescence	0.587	0.12	0.363	0.83	6	1.1e+03	0.585	0.083	0.423	0.748
Lifespan	0.190	0.11	-0.025	0.42	38	2.5e+01	0.188	0.076	0.038	0.337
Mating Frequency	0.333	0.12	0.113	0.57	11	1.4e+02	0.330	0.080	0.173	0.487
Mating Latency	0.722	0.12	0.499	0.96	13	8.0e+03	0.720	0.080	0.563	0.877
Mating Success	-0.083	0.11	-0.303	0.15	39	2.4e-01	-0.085	0.078	-0.238	0.068
Strength	0.212	0.17	-0.111	0.53	2	1.0e+01	0.211	0.145	-0.074	0.496
Ejaculate Quality and Production	0.308	0.12	0.066	0.55	23	9.4e+01	0.308	0.088	0.137	0.480
Both Reproductive Success	0.141	0.13	-0.101	0.40	12	8.0e+00	0.140	0.095	-0.046	0.327
Extinction Rate	0.348	0.20	-0.053	0.73	4	2.3e+01	0.350	0.185	-0.012	0.712
Female Reproductive Success	0.170	0.11	-0.044	0.40	102	1.8e+01	0.168	0.074	0.022	0.314
Offspring Viability	0.173	0.11	-0.041	0.40	56	1.9e+01	0.171	0.075	0.024	0.318

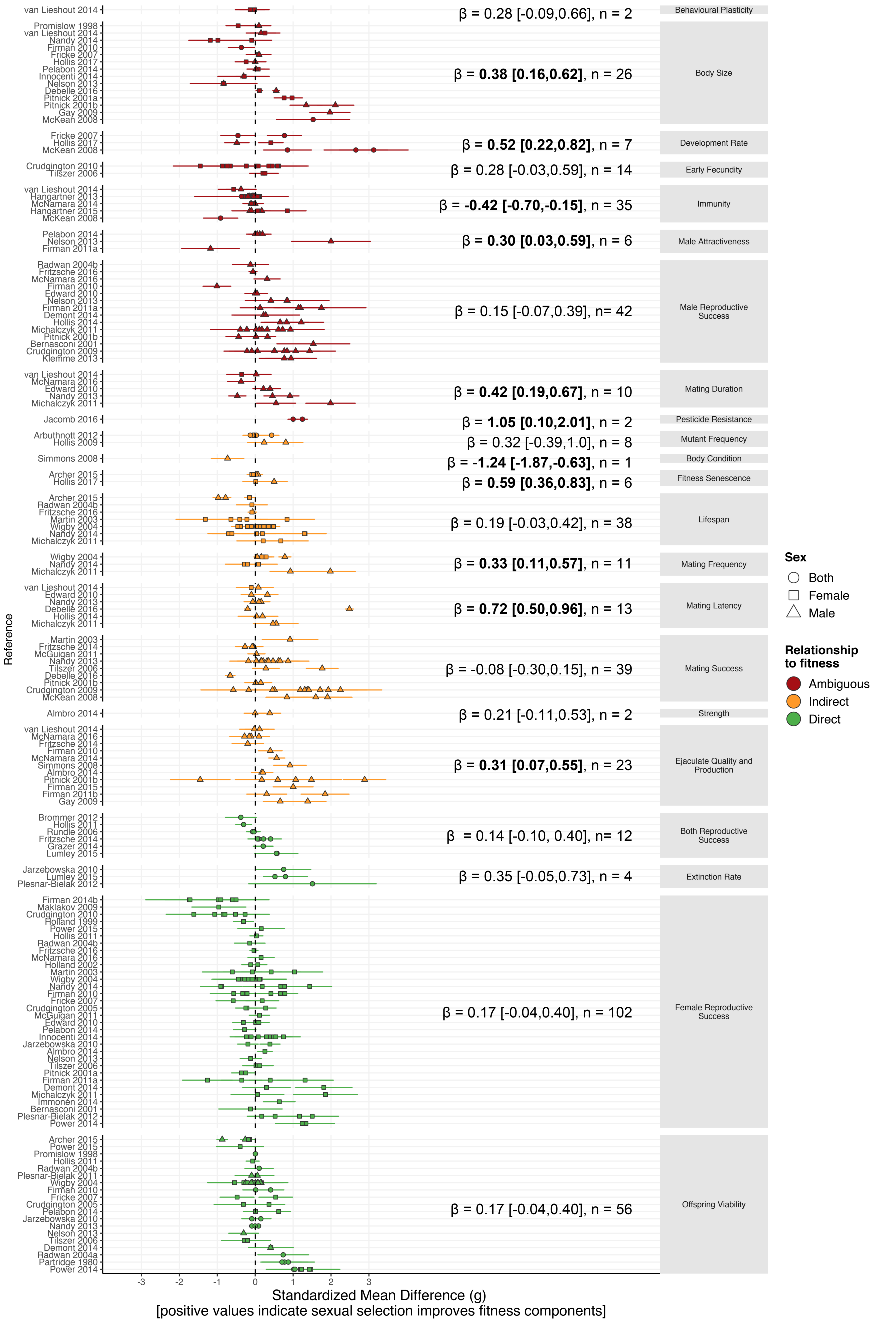

Supplementary Figure 1

Forest plot of raw effect sizes and their 95% confidence intervals, grouped according to measured fitness components and the sex of the individuals whose fitness trait was measured (male, female, or both sexes mixed together). Rows with multiple data points denote studies that provided multiple effect sizes. Positive values indicate fitness benefits of sexual selection.

Supplementary Table 8

In some instances it may be beneficial to the reader to obtain average effect sizes of each fitness trait **entirely independently** of other traits. For this reason we present a summary of independent model estimates for 16 fitness components with a sample size greater than 3 effect sizes (n>3). Unlike above where estimates were generated based on predictions from a single model, here we run individual meta-analyses for each fitness related trait. Independent models generally reduce the power and significance of some of the estimates with 'Extinction rate' and 'Ejaculate quality and production' the only two traits with p-values < 0.05.

Outcome	beta	zval	ci.lb	ci.ub	n	pval	I2	Class
Body Size	0.28	1.2	-0.16	0.72	26	0.22	97	Ambiguous
Development Rate	0.66	1.1	-0.53	1.80	7	0.28	95	Ambiguous
Early Fecundity	0.00	-0.0095	-0.45	0.44	14	0.99	49	Ambiguous
Immunity	-0.29	-1.3	-0.73	0.15	35	0.19	90	Ambiguous
Male Attractiveness	0.27	0.3	-1.50	2.00	6	0.76	99	Ambiguous
Male Reproductive Success	0.30	2.1	0.02	0.58	42	0.037	84	Ambiguous
Mating Duration	0.23	0.94	-0.25	0.70	10	0.35	91	Ambiguous
Mutant Frequency	0.25	1.1	-0.21	0.71	8	0.29	88	Indirect
Fitness Senescence	0.10	0.75	-0.15	0.35	6	0.45	81	Indirect
Lifespan	-0.08	-0.71	-0.29	0.13	38	0.48	89	Indirect
Mating Frequency	0.69	1.1	-0.57	1.90	11	0.29	99	Indirect
Mating Latency	0.28	1.9	-0.01	0.58	13	0.057	90	Indirect
Mating Success	0.39	1.8	-0.04	0.82	39	0.073	93	Indirect
Ejaculate Quality and Production	0.50	3.6	0.23	0.77	23	0.00031	83	Indirect
Both Reproductive Success	0.10	0.6	-0.24	0.45	12	0.55	87	Direct
Extinction Rate	0.62	4.9	0.37	0.87	4	9.4e-07	0	Direct
Female Reproductive Success	0.07	0.9	-0.08	0.23	102	0.37	82	Direct
Offspring Viability	0.13	1.5	-0.04	0.31	56	0.14	94	Direct

Supplementary Table 9

Bayesian model results for a preliminary model that explores many covariates collected in the dataset.

Model Parameter	Estimate	Est.Error	Q2.5	Q97.5	
b__Intercept	0.395	0.251	-0.095	0.899	
b__SexF	0.122	0.046	0.031	0.211	*
b__SexM	0.112	0.044	0.025	0.198	*
b__EnvironmentNotStated	0.028	0.146	-0.262	0.315	
b__EnvironmentStressed	0.03	0.06	-0.088	0.146	
b__logGenerations	-0.034	0.048	-0.127	0.061	
b__BlindingNotBlind	-0.065	0.201	-0.463	0.327	
b__Enforced.MonogamyYES	-0.133	0.086	-0.301	0.034	
b__SexF:EnvironmentNotStated	0.122	0.127	-0.13	0.374	
b__SexM:EnvironmentNotStated	0.076	0.119	-0.16	0.316	
b__SexF:EnvironmentStressed	0.09	0.067	-0.039	0.223	
b__SexM:EnvironmentStressed	-0.132	0.068	-0.264	0.002	
sd__Outcome___Intercept	0.327	0.074	0.214	0.499	*
sd__Study.ID___Intercept	0.485	0.053	0.393	0.6	*
sd__Taxon___Intercept	0.134	0.121	0.005	0.439	*

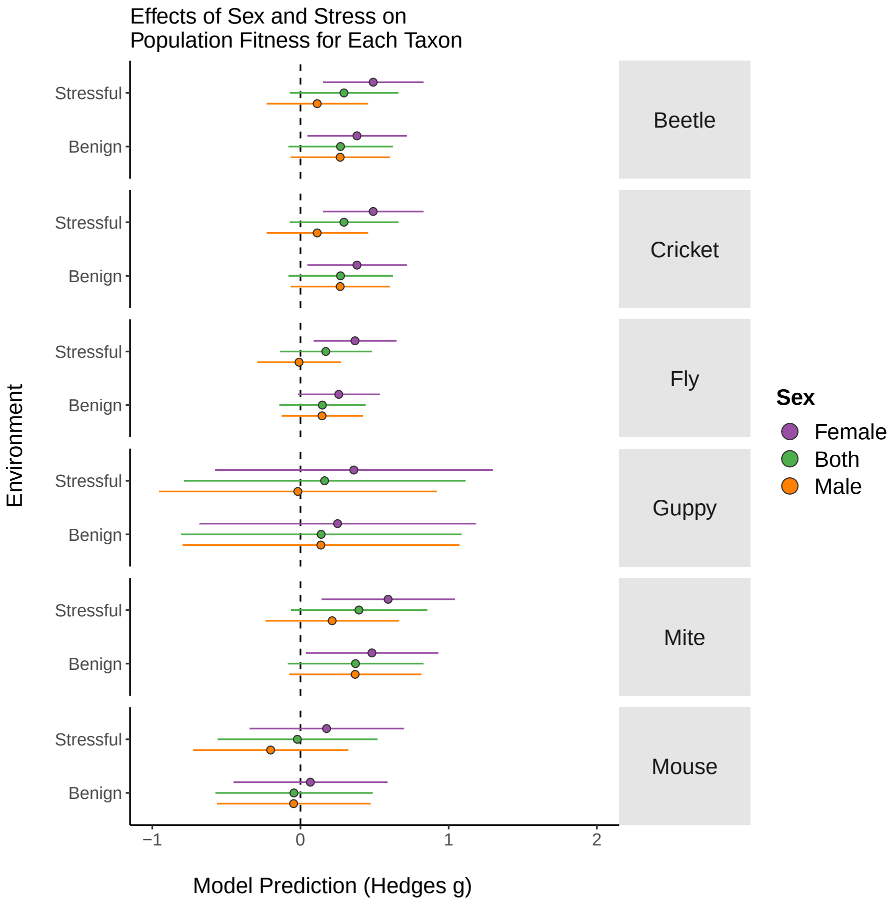

Supplementary Figure 2

The predictions from this model indicate some heterogeneity between taxon. However, the most apparent difference between taxa is that confidence bands increase for taxa with low sample size. As previously shown, the beetle and fly taxa are the most heavily sampled and in the above figure have the narrowest confidence bands. Importantly, the overall direction of effect does not change between taxon, although guppies and mice show near zero effect sizes. Here we see that under stressed environments, females from all taxa appear to have greater fitness increase than males or 'both'.

Supplementary Table 10

The predictions for the above figure looking at the effect of sexual selection amongst taxa uses the following dataframe.

Sex	Environment	Taxon	Prediction	SE	CI.lb	CI.ub	n
M	Unstressed	Beetle	0.2680	0.17	-0.066	0.60	18
B	Unstressed	Beetle	0.2702	0.18	-0.081	0.62	2
F	Unstressed	Beetle	0.3810	0.17	0.047	0.71	15
M	Stressed	Beetle	0.1128	0.17	-0.228	0.45	2
B	Stressed	Beetle	0.2934	0.19	-0.072	0.66	6
F	Stressed	Beetle	0.4905	0.17	0.153	0.83	9
M	Unstressed	Cricket	0.2680	0.17	-0.066	0.60	1
B	Unstressed	Cricket	0.2702	0.18	-0.081	0.62	NA
F	Unstressed	Cricket	0.3810	0.17	0.047	0.71	NA
M	Stressed	Cricket	0.1128	0.17	-0.228	0.45	NA
B	Stressed	Cricket	0.2934	0.19	-0.072	0.66	NA
F	Stressed	Cricket	0.4905	0.17	0.153	0.83	NA
M	Unstressed	Fly	0.1453	0.14	-0.128	0.42	60
B	Unstressed	Fly	0.1475	0.15	-0.142	0.44	9
F	Unstressed	Fly	0.2583	0.14	-0.017	0.53	93
M	Stressed	Fly	-0.0099	0.14	-0.290	0.27	9
B	Stressed	Fly	0.1707	0.16	-0.138	0.48	8
F	Stressed	Fly	0.3678	0.14	0.090	0.65	19
M	Unstressed	Guppy	0.1374	0.48	-0.796	1.07	NA
B	Unstressed	Guppy	0.1395	0.48	-0.806	1.08	NA
F	Unstressed	Guppy	0.2503	0.48	-0.682	1.18	3
M	Stressed	Guppy	-0.0178	0.48	-0.953	0.92	NA
B	Stressed	Guppy	0.1627	0.48	-0.786	1.11	NA
F	Stressed	Guppy	0.3599	0.48	-0.575	1.30	NA
M	Unstressed	Mite	0.3690	0.23	-0.075	0.81	3
B	Unstressed	Mite	0.3711	0.23	-0.085	0.83	2
F	Unstressed	Mite	0.4820	0.23	0.037	0.93	9
M	Stressed	Mite	0.2138	0.23	-0.235	0.66	1
B	Stressed	Mite	0.3943	0.23	-0.064	0.85	4
F	Stressed	Mite	0.5915	0.23	0.143	1.04	3
M	Unstressed	Mouse	-0.0463	0.26	-0.564	0.47	1
B	Unstressed	Mouse	-0.0442	0.27	-0.574	0.49	2
F	Unstressed	Mouse	0.0666	0.26	-0.452	0.58	5
M	Stressed	Mouse	-0.2016	0.27	-0.723	0.32	NA
B	Stressed	Mouse	-0.0210	0.27	-0.558	0.52	NA
F	Stressed	Mouse	0.1761	0.27	-0.344	0.70	5

Supplementary Table 11

Using the `anova.rma` function we can conduct hypothesis tests between two categorical groups in the model. Here we conduct 5 tests comparing the relative effect of sexual selection between the sexes, and in different environments.

	Estimate	Est.Error	CI.Lower	CI.Upper	
**M vs F, Benign**	-0.113	0.030	-0.173	-0.054	
**M vs F, Stressful**	-0.377	0.046	-0.468	-0.287	*
**Benign vs Stressful, Female**	-0.108	0.037	-0.181	-0.036	*
**Benign vs Stressful, Male**	0.156	0.043	0.072	0.240	*
**Benign vs Stressful, Both**	-0.028	0.080	-0.184	0.128	

Supplementary Table 12

Model estimate summary table for the Bayesian model investigating the effect of environment and sex (alongside sexual selection) on fitness.

Model Parameter	Estimate	Est.Error	Q2.5	Q97.5	
b_Intercept	0.188	0.175	-0.167	0.522	
b_SexB	0.003	0.073	-0.143	0.144	
b_SexF	0.113	0.03	0.053	0.171	*
b_EnvironmentStressed	-0.156	0.043	-0.241	-0.073	*
b_SexB:EnvironmentStressed	0.182	0.089	0.006	0.354	*
b_SexF:EnvironmentStressed	0.264	0.052	0.163	0.369	*
sd_Outcome___Intercept	0.413	0.119	0.241	0.703	*
sd_Study.ID___Intercept	0.452	0.053	0.36	0.57	*
sd_Taxon___Intercept	0.147	0.152	0.004	0.545	*

Supplementary Table 13

Hypothesis tests for the Bayesian model are similar to the REML model, with slight differences to CIs.

	Estimate	Est.Error	CI.Lower	CI.Upper	
**M vs F, Benign**	-0.113	0.030	-0.171	-0.053	*
**M vs F, Stressful**	-0.377	0.047	-0.471	-0.286	*
**Benign vs Stressful, Female**	-0.109	0.037	-0.182	-0.035	*
**Benign vs Stressful, Male**	0.156	0.043	0.073	0.241	*
**Benign vs Stressful, Both**	-0.026	0.080	-0.182	0.133	

Supplementary Table 14

The REML predictions in Figure 2a within the manuscript use the following dataframe

Sex	Environment	Prediction	SE	CI.lb	CI.ub	n
Male	Benign	0.188	0.12	-0.053	0.43	83
Both	Benign	0.191	0.13	-0.071	0.45	15
Female	Benign	0.301	0.12	0.058	0.54	125
Male	Stressful	0.032	0.13	-0.218	0.28	12
Both	Stressful	0.219	0.14	-0.061	0.5	18
Female	Stressful	0.409	0.13	0.162	0.66	36

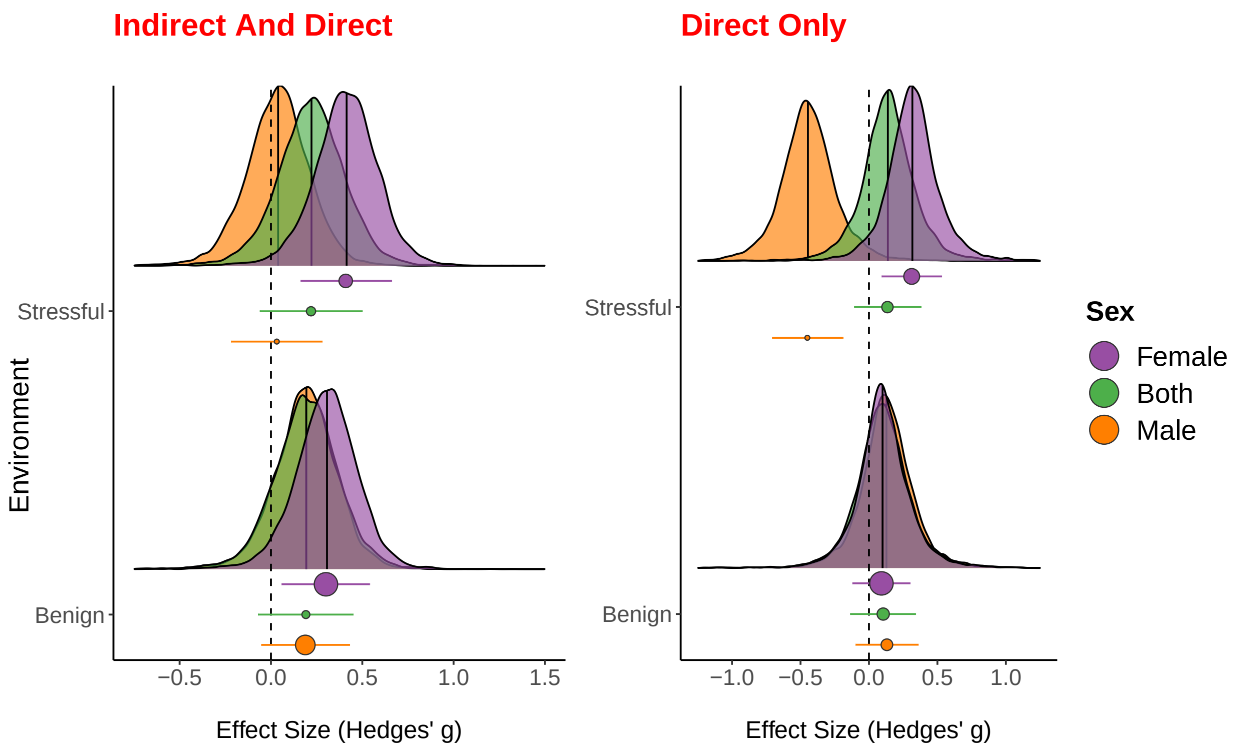

Supplementary Figure 3

The comparison of model predictions between models that use a dataset compiled from direct **and** indirect fitness components against a model that only uses direct fitness components. Notably, in both cases there is a significant positive effect of sexual selection on fitness for females evolving in stressful conditions. Bayesian predictions are depicted by a density curve while REML predictions are depicted with a circle (size corresponds to n) and 95 % CIs.

Supplementary Table 15

The REML predictions for the 'direct model' in the plot above use the following dataframe.

Sex	Environment	Prediction	SE	CI.lb	CI.ub	n
Male	Benign	0.131	0.12	-0.098	0.36	13
Both	Benign	0.104	0.12	-0.137	0.34	15
Female	Benign	0.091	0.11	-0.121	0.3	86
Male	Stressful	-0.45	0.13	-0.707	-0.19	2
Both	Stressful	0.135	0.12	-0.108	0.38	12
Female	Stressful	0.312	0.11	0.093	0.53	31

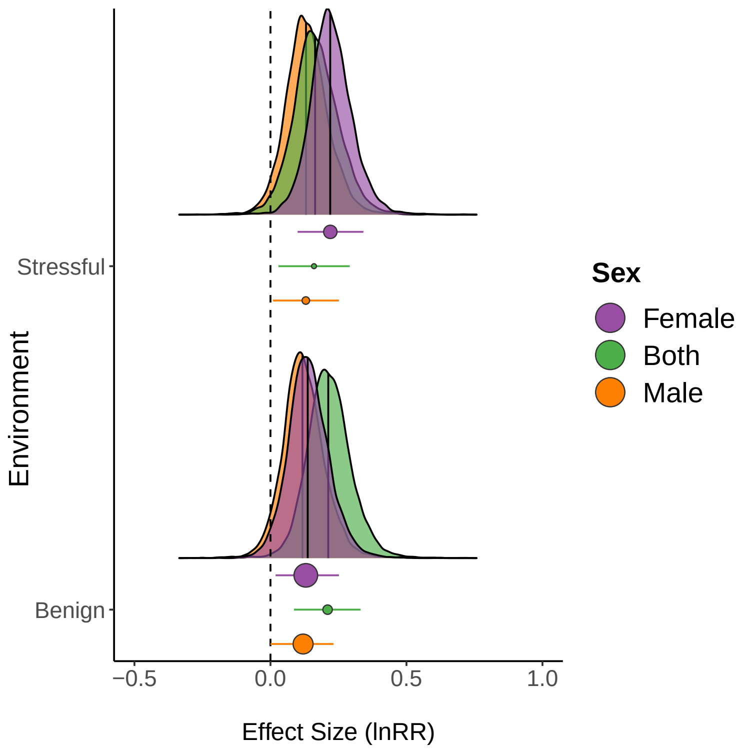

Supplementary Figure 4

Using an alternative effect size (lnRR) sexual selection also increases population fitness. Circle size is proportional to sample size (shown below). The REML predictions are shown as circles with error bars and the Bayesian predictions as density ridges. Note that the magnitude of the effect sizes presented here should not be directly compared with those using Hedges' g as lnRR is a log-transformed value.

Supplementary Table 16

The REML predictions for the meta-analysis using lnRR (plotted above) are formulated from the following model predictions.

Sex	Environment	Prediction	SE	CI.lb	CI.ub	n
Male	Benign	0.12	0.059	7e-05	0.23	73
Both	Benign	0.21	0.063	0.08705	0.33	12
Female	Benign	0.13	0.059	0.0193	0.25	110
Male	Stressful	0.13	0.061	0.00907	0.25	8
Both	Stressful	0.16	0.068	0.02938	0.29	6
Female	Stressful	0.22	0.060	0.1002	0.34	27

Supplementary Table 17

Model estimates, including random effect sigma value for the model of phenotypic variance (lnCVR)

Model Parameter	Estimate	Est.Error	Q2.5	Q97.5	
b_Intercept	0.067	0.304	-0.537	0.671	
b_SexB	-0.257	0.039	-0.333	-0.181	*
b_SexF	-0.031	0.015	-0.06	-0.002	*
b_EnvironmentStressed	0.156	0.022	0.115	0.199	*
b_SexB:EnvironmentStressed	-0.733	0.044	-0.82	-0.645	*
b_SexF:EnvironmentStressed	-0.975	0.026	-1.027	-0.925	*
sd_Outcome___Intercept	0.557	0.16	0.342	0.963	*
sd_Study.ID___Intercept	0.267	0.038	0.203	0.352	*
sd_Taxon___Intercept	0.521	0.32	0.137	1.319	*

Supplementary Table 18

The REML predictions in figure 2b within the manuscript uses the following dataframe (model predictions).

Sex	Environment	Prediction	SE	CI.lb	CI.ub	n
Male	Benign	0.068	0.23	-0.38	0.51	73
Both	Benign	-0.187	0.23	-0.64	0.26	12
Female	Benign	0.037	0.23	-0.41	0.48	110
Male	Stressful	0.225	0.23	-0.22	0.67	8
Both	Stressful	-0.764	0.23	-1.22	-0.31	6
Female	Stressful	-0.781	0.23	-1.23	-0.34	27

Supplementary Table 19

Bayesian hypothesis tests between categorical groups for phenotypic variation (lnCVR).

	Estimate	Est.Error	CI.Lower	CI.Upper	
**M vs F, Benign**	0.031	0.015	0.0016	0.06	*
**M vs F, Stressful**	1.006	0.024	0.9600	1.05	*
**Benign vs Stressful, Female**	0.819	0.018	0.7828	0.85	*
**Benign vs Stressful, Male**	-0.156	0.022	-0.1990	-0.11	*
**Benign vs Stressful, Both**	0.577	0.040	0.4978	0.65	*

Supplementary Table 20

REML hypothesis tests between categorical groups for phenotypic variation (lnCVR).

	Estimate	Est.Error	CI.Lower	CI.Upper	
**M vs F, Benign**	0.031	0.015	0.0025	0.06	*
**M vs F, Stressful**	1.006	0.024	0.9598	1.05	*
**Benign vs Stressful, Female**	0.818	0.018	0.7831	0.85	*
**Benign vs Stressful, Male**	-0.157	0.022	-0.1990	-0.11	*
**Benign vs Stressful, Both**	0.577	0.040	0.4995	0.65	*

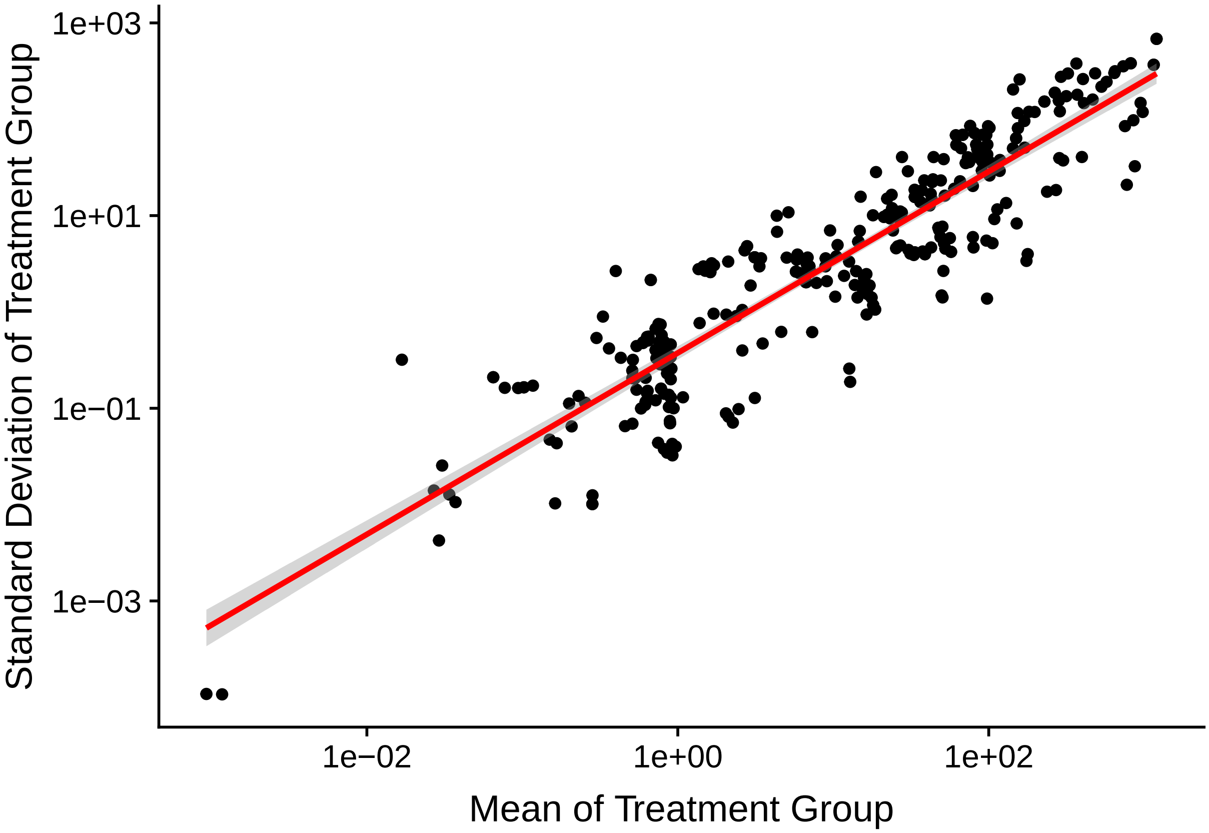

Supplementary Figure 5

The use of lnCVR (as opposed to lnVR) is justified in this meta-analysis due to the strong mean-variance relationship. In this case the standard deviation from the treatment group is compared to the means of the treatment group on a log-scale.

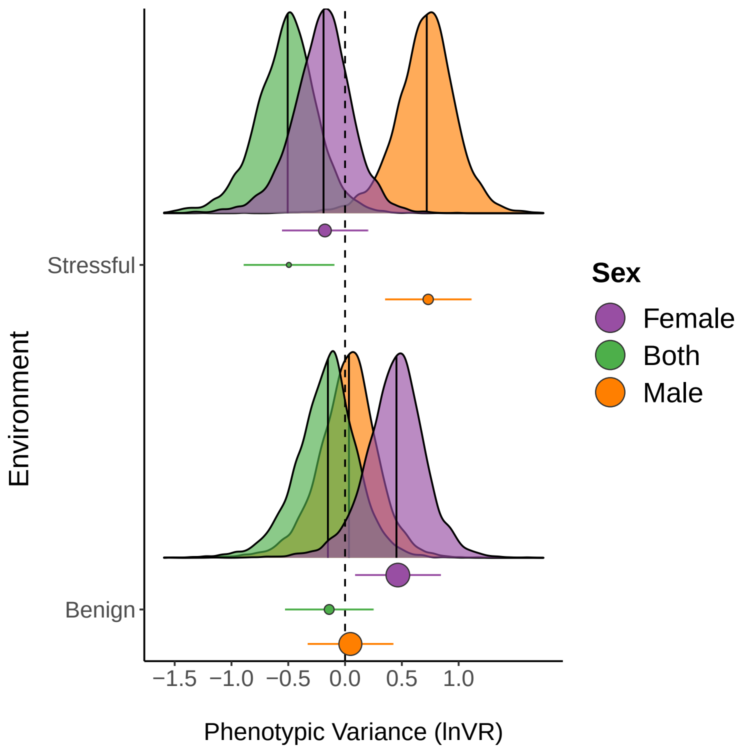

Supplementary Figure 6

The effects of sexual selection on the log-variance ratio (lnVR). Without accounting for the mean variance relationship (lnCVR) sexual selection has different effects on the variance measure.

Supplementary Table 21

The points in the above plot are based on the following REML model and predictions. While the predicted effects of sexual selection on lnVR in stressful conditions is non-significantly negative, there is still a negative significant interaction between stressful environments and the female sex.

Sex	Environment	Prediction	SE	CI.lb	CI.ub	n
Male	Benign	0.047	0.19	-0.33	0.42	132
Both	Benign	-0.14	0.2	-0.53	0.25	17
Female	Benign	0.46	0.19	0.09	0.84	142
Male	Stressful	0.73	0.19	0.35	1.1	19
Both	Stressful	-0.49	0.2	-0.89	-0.098	8
Female	Stressful	-0.18	0.19	-0.55	0.2	31

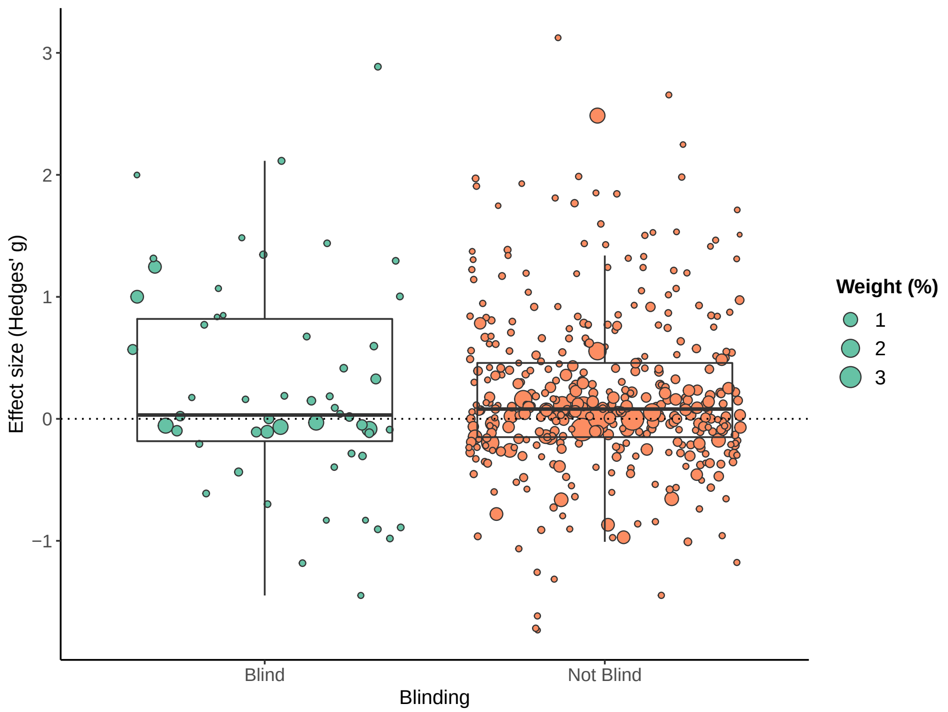

Supplementary Figure 7

Blinding does not appear to alter the magnitude or direction of effect sizes for the studies used in this meta-analysis. However, this should not be viewed as evidence against the validity of blinding as a research method.

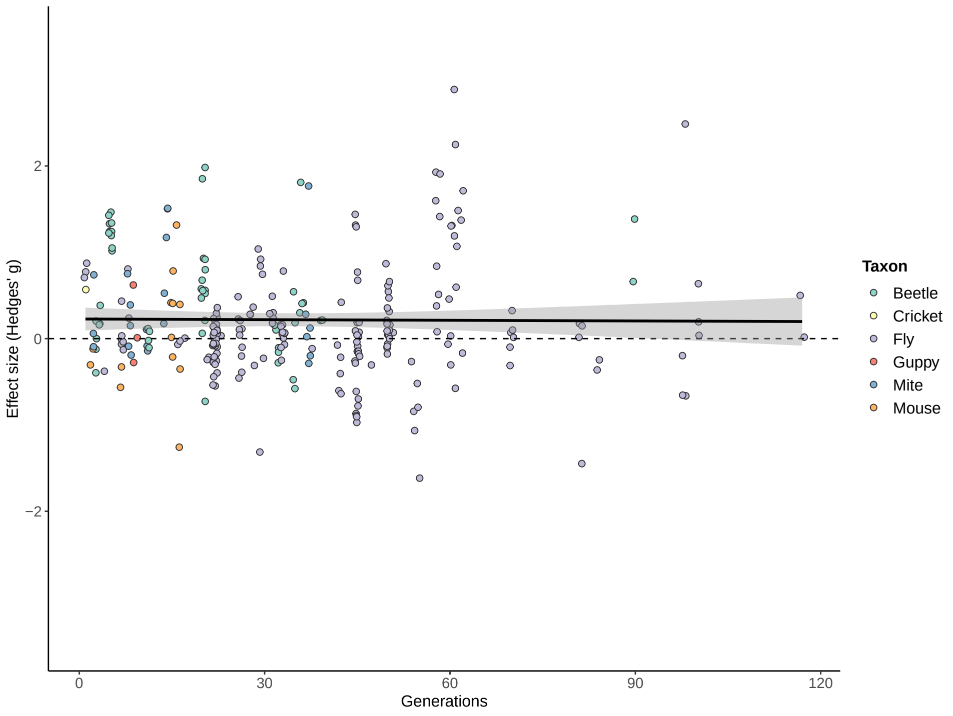

Supplementary Figure 8

The number of generations an experimental evolution procedure is run for does not appear to affect the magnitude or direction of the effect size from the fitness related outcome measured at that point.

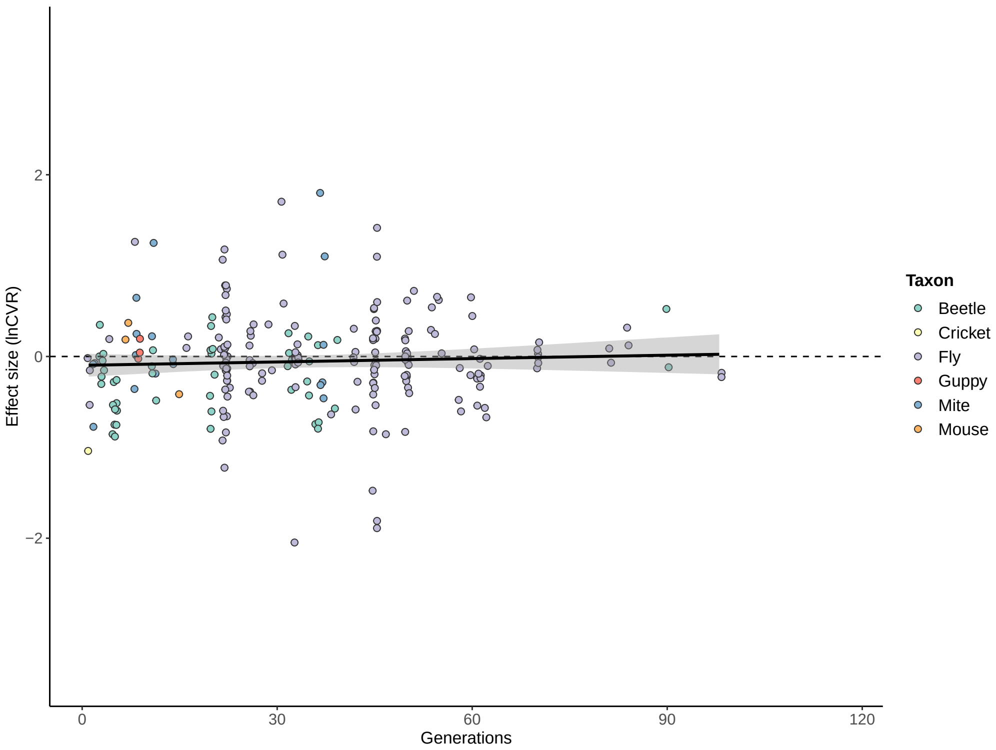

Supplementary Figure 9

Phenotypic variation (lnCVR) is not affected by the number of generations an experiment is ran for.

Supplementary References

1. Partridge, L. Mate choice increases a component of offspring fitness in fruit-flies. *Nature* **283**, 290–291 (1980).

2. Price, T. A. R., Hurst, G. D. D. & Wedell, N. Polyandry prevents extinction. *Current Biology* **20**, 471–475 (2010).

3. Savic Veselinovic, M., Pavkovic-Lucic, S., Kurbalija Novicic, Z., Jelic, M. & Andelkovic, M. Sexual selection can reduce mutational load in drosophila subobscura. *Genetika-Belgrade* **45**, 537–552 (2013).

4. Re, A. C. D. *Compute.es: Compute effect sizes. R Package* (2013).

5. Lieshout, E. van, McNamara, K. B. & Simmons, L. W. Rapid loss of behavioral plasticity and immunocompetence under intense sexual selection. *Evolution* **68**, 2550–2558 (2014).

6. Simmons, L. W. & Garcia-Gonzalez, F. Evolutionary reduction in testes size and competitive fertilization success in response to the experimental removal of sexual selection in dung beetles. *Evolution* **62**, 2580–2591 (2008).

7. Almbro, M. & Simmons, L. W. Sexual selection can remove an experimentally induced mutation load. *Evolution* **68**, 295–300 (2014).

8. Fricke, C. & Arnqvist, G. Rapid adaptation to a novel host in a seed beetle (callosobruchus maculatus): The role of sexual selection. *Evolution* **61**, 440–454 (2007).

9. Hollis, B. & Kawecki, T. J. Male cognitive performance declines in the absence of sexual selection. *Proceedings of the Royal Society B-Biological Sciences* **281**, (2014).

10. McKean, K. A. & Nunney, L. Sexual selection and immune function in drosophila melanogaster. *Evolution* **62**, 386–400 (2008).

11. Crudgington, H. S., Fellows, S. & Snook, R. R. Increased opportunity for sexual conflict promotes harmful males with elevated courtship frequencies. *Journal of Evolutionary Biology* **23**, 440–446 (2010).

12. Tilszer, M., Antoszczyk, K., Sałek, N., Zajac, E. & Radwan, J. Evolution under relaxed sexual conflict in the bulb mite rhizoglyphus robini. *Evolution* **60**, 1868–1873 (2006).

13. Hangartner, S., Michalczyk, L., Gage, M. J. G. & Martin, O. Y. Experimental removal of sexual selection leads to decreased investment in an immune component in female tribolium castaneum. *Infection, Genetics and Evolution* **33**, 212–218 (2015).

14. Hangartner, S., Sbilordo, S. H., Michalczyk, L., Gage, M. J. G. & Martin, O. Y. Are there genetic trade-offs between immune and reproductive investments in tribolium castaneum? *Infection, Genetics and Evolution* **19**, 45–50 (2013).

15. McNamara, K. B., Lieshout, E. van & Simmons, L. W. A test of the sexy-sperm and good-sperm hypotheses for the evolution of polyandry. *Behavioral Ecology* **25**, 989–995 (2014).

16. Firman, R. C. Female social preference for males that have evolved via monogamy: Evidence of a trade-off between pre- and post-copulatory sexually selected traits? *Biology Letters* **10**, (2014).

17. Nelson, A. C., Colson, K. E., Harmon, S. & Potts, W. K. Rapid adaptation to mammalian sociality via sexually selected traits. *Bmc Evolutionary Biology* **13**, (2013).

18. Pélabon, C. *et al.* The effects of sexual selection on life-history traits: An experimental study on guppies. *Journal of Evolutionary Biology* **27**, 404–416 (2014).

19. Edward, D. A., Fricke, C. & Chapman, T. Adaptations to sexual selection and sexual conflict: Insights from experimental evolution and artificial selection. *Philosophical Transactions of the Royal Society B-Biological*

Sciences **365**, 2541–2548 (2010).

20. Michalczyk, L. *et al.* Experimental evolution exposes female and male responses to sexual selection and conflict in tribolium castaneum. *Evolution* **65**, 713–724 (2011).

21. Nandy, B., Chakraborty, P., Gupta, V., Ali, S. Z. & Prasad, N. G. Sperm competitive ability evolves in response to experimental alteration of operational sex ratio. *Evolution* **67**, 2133–2141 (2013).

22. Jacomb, F., Marsh, J. & Holman, L. Sexual selection expedites the evolution of pesticide resistance. *Evolution* **70**, 2746–2751 (2016).

23. Arbuthnott, D. & Rundle, H. D. Sexual selection is ineffectual or inhibits the purging of deleterious mutations in drosophila melanogaster. *Evolution* **66**, 2127–2137 (2012).

24. Hollis, B., Fierst, J. L. & Houle, D. Sexual selection accelerates the elimination of a deleterious mutant in drosophila melanogaster. *Evolution* **63**, 324–333 (2009).

25. Archer, C. R. *et al.* Sex-specific effects of natural and sexual selection on the evolution of life span and ageing in drosophila simulans. *Functional Ecology* **29**, 562–569 (2015).

26. Wigby, S. & Chapman, T. Female resistance to male harm evolves in response to manipulation of sexual conflict. *Evolution* **58**, 1028–1037 (2004).

27. Martin, O. Y. & Hosken, D. J. Costs and benefits of evolving under experimentally enforced polyandry or monogamy. *Evolution* **57**, 2765–2772 (2003).

28. Debelle, A., Ritchie, M. G. & Snook, R. R. Sexual selection and assortative mating: An experimental test. *Journal of Evolutionary Biology* **29**, 1307–1316 (2016).

29. McGuigan, K., Petfield, D. & Blows, M. W. Reducing mutation load through sexual selection on males. *Evolution* **65**, 2816–2829 (2011).

30. Crudgington, H. S., Fellows, S., Badcock, N. S. & Snook, R. R. Experimental manipulation of sexual selection promotes greater male mating capacity but does not alter sperm investment. *Evolution* **63**, 926–938 (2009).

31. Firman, R. C. & Simmons, L. W. Experimental evolution of sperm quality via postcopulatory sexual selection in house mice. *Evolution* **64**, 1245–1256 (2010).

32. Fritzsche, K., Timmermeyer, N., Wolter, M. & Michiels, N. K. Female, but not male, nematodes evolve under experimental sexual coevolution. *Proceedings of the Royal Society B-Biological Sciences* **281**, (2014).

33. Gay, L., Hosken, D. J., Vasudev, R., Tregenza, T. & Eady, P. E. Sperm competition and maternal effects differentially influence testis and sperm size in callosobruchus maculatus. *Journal of Evolutionary Biology* **22**, 1143–1150 (2009).

34. McNamara, K. B. *et al.* Male-biased sex ratio does not promote increased sperm competitiveness in the seed beetle, callosobruchus maculatus. *Scientific Reports* **6**, (2016).

35. Jarzebowska, M. & Radwan, J. Sexual selection counteracts extinction of small populations of the bulb mites. *Evolution* **64**, 1283–1289 (2010).

36. Plesnar-Bielak, A., Skrzynecka, A. M., Prokop, Z. M. & Radwan, J. Mating system affects population performance and extinction risk under environmental challenge. *Proceedings of the Royal Society B-Biological Sciences* **279**, 4661–4667 (2012).

37. Lumley, A. J. *et al.* Sexual selection protects against extinction. *Nature* **522**, 470–+ (2015).

38. Plesnar, A., Konior, M. & Radwan, J. The role of sexual selection in purging the genome of induced mutations in the bulb mite (rizoglyphus robini). *Evolutionary Ecology Research* **13**, 209–216 (2011).

39. Firman, R. C. Polyandrous females benefit by producing sons that achieve high reproductive success in a

competitive environment. *Proceedings of the Royal Society B-Biological Sciences* **278**, 2823–2831 (2011).

40. Bernasconi, G. & Keller, L. Female polyandry affects their sons' reproductive success in the red flour beetle tribolium castaneum. *Journal of Evolutionary Biology* **14**, 186–193 (2001).

41. Aguirre, J. D. & Marshall, D. J. Does genetic diversity reduce sibling competition? *Evolution* **66**, 94–102 (2012).

42. Ahuja, A. & Singh, R. S. Variation and evolution of male sex combs in drosophila: Nature of selection response and theories of genetic variation for sexual traits. *Genetics* **179**, 503–509 (2008).

43. Amitin, E. G. & Pitnick, S. Influence of developmental environment on male- and female-mediated sperm precedence in drosophila melanogaster. *Journal of Evolutionary Biology* **20**, 381–391 (2007).

44. Antolin, M. F., Ode, P. J., Heimpel, G. E., O'Hara, R. B. & Strand, M. R. Population structure, mating system, and sex-determining allele diversity of the parasitoid wasp habrobracon hebetor. *Heredity* **91**, 373–381 (2003).

45. Arbuthnott, D., Dutton, E. M., Agrawal, A. F. & Rundle, H. D. The ecology of sexual conflict: Ecologically dependent parallel evolution of male harm and female resistance in drosophila melanogaster. *Ecology Letters* **17**, 221–228 (2014).

46. Arbuthnott, D. & Rundle, H. D. Misalignment of natural and sexual selection among divergently adapted drosophila melanogaster populations. *Animal Behaviour* **87**, 45–51 (2014).

47. Artieri, C. G., Haerty, W., Gupta, B. P. & Singh, R. S. Sexual selection and maintenance of sex: Evidence from comparisons of rates of genomic accumulation of mutations and divergence of sex-related genes in sexual and hermaphroditic species of caenorhabditis. *Molecular Biology and Evolution* **25**, 972–979 (2008).

48. Bacigalupe, L. D., Crudgington, H. S., Hunter, F., Moore, A. J. & Snook, R. R. Sexual conflict does not drive reproductive isolation in experimental populations of drosophila pseudoobscura. *Journal of Evolutionary Biology* **20**, 1763–1771 (2007).

49. Bacigalupe, L. D., Crudgington, H. S., Slate, J., Moore, A. J. & Snook, R. R. Sexual selection and interacting phenotypes in experimental evolution: A study of drosophila pseudoobscura mating behavior. *Evolution* **62**, 1804–1812 (2008).

50. Barbosa, M., Connolly, S. R., Hisano, M., Dornelas, M. & Magurran, A. E. Fitness consequences of female multiple mating: A direct test of indirect benefits. *Bmc Evolutionary Biology* **12**, (2012).

51. Bielak, A. P., Skrzynecka, A. M., Miler, K. & Radwan, J. Selection for alternative male reproductive tactics alters intralocus sexual conflict. *Evolution* **68**, 2137–2144 (2014).

52. Blows, M. W. Interaction between natural and sexual selection during the evolution of mate recognition. *Proceedings of the Royal Society B-Biological Sciences* **269**, 1113–1118 (2002).

53. Brommer, J. E., Fricke, C., Edward, D. A. & Chapman, T. Interactions between genotype and sexual conflict environment influence transgenerational fitness in drosophila melanogaster. *Evolution* **66**, 517–531 (2012).

54. Castillo, D. M., Burger, M. K., Lively, C. M. & Delph, L. F. Experimental evolution: Assortative mating and sexual selection, independent of local adaptation, lead to reproductive isolation in the nematode caenorhabditis remanei. *Evolution* **69**, 3141–3155 (2015).

55. Cayetano, L., Maklakov, A. A., Brooks, R. C. & Bonduriansky, R. Evolution of male and female genitalia following release from sexual selection. *Evolution* **65**, 2171–2183 (2011).

56. Chandler, C. H., Ofria, C. & Dworkin, I. Runaway sexual selection leads to good genes. *Evolution* **67**, 110–119 (2013).

57. Chenoweth, S. F., Appleton, N. C., Allen, S. L. & Rundle, H. D. Genomic evidence that sexual selection

impedes adaptation to a novel environment. *Current Biology* **25**, 1860–1866 (2015).

58. Chenoweth, S. F., Petfield, D., Doughty, P. & Blows, M. W. Male choice generates stabilizing sexual selection on a female fecundity correlate. *Journal of Evolutionary Biology* **20**, 1745–1750 (2007).

59. Chenoweth, S. F., Rundle, H. D. & Blows, M. W. Genetic constraints and the evolution of display trait sexual dimorphism by natural and sexual selection. *American Naturalist* **171**, 22–34 (2008).

60. Chenoweth, S. F., Rundle, H. D. & Blows, M. W. Experimental evidence for the evolution of indirect genetic effects: Changes in the interaction effect coefficient, psi, due to sexual selection. *Evolution* **64**, 1849–1856 (2010).

61. Crudgington, H. S., Beckerman, A. P., Brüstle, L., Green, K. & Snook, R. R. Experimental removal and elevation of sexual selection: Does sexual selection generate manipulative males and resistant females? *American Naturalist* **165**, S72–S87 (2005).

62. Demont, M. *et al.* Experimental removal of sexual selection reveals adaptations to polyandry in both sexes. *Evolutionary Biology* **41**, 62–70 (2014).

63. Fava, G. Studies on the selective agents operating in experimental populations of tisbe clodiensis (copepoda, harpacticoida). *Genetica* **45**, 289–305 (1975).

64. Firman, R. C., Cheam, L. Y. & Simmons, L. W. Sperm competition does not influence sperm hook morphology in selection lines of house mice. *Journal of Evolutionary Biology* **24**, 856–862 (2011).

65. Firman, R. C. *et al.* Evolutionary change in testes tissue composition among experimental populations of house mice. *Evolution* **69**, 848–855 (2015).

66. Firman, R. C., Gomendio, M., Roldan, E. R. S. & Simmons, L. W. The coevolution of ova defensiveness with sperm competitiveness in house mice. *American Naturalist* **183**, 565–572 (2014).

67. Firman, R. C. & Simmons, L. W. Experimental evolution of sperm competitiveness in a mammal. *Bmc Evolutionary Biology* **11**, (2011).

68. Firman, R. C. & Simmons, L. W. Male house mice evolving with post-copulatory sexual selection sire embryos with increased viability. *Ecology Letters* **15**, 42–46 (2012).

69. Fricke, C., Andersson, C. & Arnqvist, G. Natural selection hampers divergence of reproductive traits in a seed beetle. *Journal of Evolutionary Biology* **23**, 1857–1867 (2010).

70. Fritzsche, K., Booksmythe, I. & Arnqvist, G. Sex ratio bias leads to the evolution of sex role reversal in honey locust beetles. *Current Biology* **26**, 2522–2526 (2016).

71. Garcia-Gonzalez, F., Yasui, Y. & Evans, J. P. Mating portfolios: Bet-hedging, sexual selection and female multiple mating. *Proceedings of the Royal Society B-Biological Sciences* **282**, (2015).

72. Gay, L., Eady, P. E., Vasudev, R., Hosken, D. J. & Tregenza, T. Does reproductive isolation evolve faster in larger populations via sexually antagonistic coevolution? *Biology Letters* **5**, 693–696 (2009).

73. Gay, L., Hosken, D. J., Eady, P., Vasudev, R. & Tregenza, T. The evolution of harm-effect of sexual conflicts and population size. *Evolution* **65**, 725–737 (2011).

74. Grazer, V. M., Demont, M., Michalczyk, L., Gage, M. J. G. & Martin, O. Y. Environmental quality alters female costs and benefits of evolving under enforced monogamy. *Bmc Evolutionary Biology* **14**, (2014).

75. Grieshop, K., Stangberg, J., Martinossi-Allibert, I., Arnqvist, G. & Berger, D. Strong sexual selection in males against a mutation load that reduces offspring production in seed beetles. *Journal of Evolutionary Biology* **29**, 1201–1210 (2016).

76. Hall, M. D., Bussiere, L. F. & Brooks, R. Diet-dependent female evolution influences male lifespan in a nuptial feeding insect. *Journal of Evolutionary Biology* **22**, 873–881 (2009).

77. Hicks, S. K., Hagenbuch, K. L. & Meffert, L. M. Variable costs of mating, longevity, and starvation

resistance in musca domestica (diptera: Muscidae). *Environmental Entomology* **33**, 779–786 (2004).

78. Holland, B. Sexual selection fails to promote adaptation to a new environment. *Evolution* **56**, 721–730 (2002).

79. Holland, B. & Rice, W. R. Experimental removal of sexual selection reverses intersexual antagonistic coevolution and removes a reproductive load. *Proceedings of the National Academy of Sciences of the United States of America* **96**, 5083–5088 (1999).

80. Hollis, B. & Houle, D. Populations with elevated mutation load do not benefit from the operation of sexual selection. *Journal of Evolutionary Biology* **24**, 1918–1926 (2011).

81. Hollis, B., Houle, D. & Kawecki, T. J. Evolution of reduced post-copulatory molecular interactions in drosophila populations lacking sperm competition. *Journal of Evolutionary Biology* **29**, 77–85 (2016).

82. Hollis, B., Houle, D., Yan, Z., Kawecki, T. J. & Keller, L. Evolution under monogamy feminizes gene expression in drosophila melanogaster. *Nature Communications* **5**, (2014).

83. Hollis, B., Keller, L. & Kawecki, T. J. Sexual selection shapes development and maturation rates in drosophila. *Evolution* **71**, 304–314 (2017).

84. Hosken, D. J., Martin, O. Y., Wigby, S., Chapman, T. & Hodgson, D. J. Sexual conflict and reproductive isolation in flies. *Biology Letters* **5**, 697–699 (2009).

85. House, C. M. *et al.* Sexual and natural selection both influence male genital evolution. *Plos One* **8**, (2013).

86. Hunt, J., Snook, R. R., Mitchell, C., Crudgington, H. S. & Moore, A. J. Sexual selection and experimental evolution of chemical signals in drosophila pseudoobscura. *Journal of Evolutionary Biology* **25**, 2232–2241 (2012).

87. Immonen, E., Snook, R. R. & Ritchie, M. G. Mating system variation drives rapid evolution of the female transcriptome in drosophila pseudoobscura. *Ecology and Evolution* **4**, 2186–2201 (2014).

88. Innocenti, P., Flis, I. & Morrow, E. H. Female responses to experimental removal of sexual selection components in drosophila melanogaster. *Bmc Evolutionary Biology* **14**, (2014).

89. Janicke, T., Sandner, P., Ramm, S. A., Vizoso, D. B. & Schaerer, L. Experimentally evolved and phenotypically plastic responses to enforced monogamy in a hermaphroditic flatworm. *Journal of Evolutionary Biology* **29**, 1713–1727 (2016).

90. Klemme, I. & Firman, R. C. Male house mice that have evolved with sperm competition have increased mating duration and paternity success. *Animal Behaviour* **85**, 751–758 (2013).

91. Long, T. A. F., Agrawal, A. F. & Rowe, L. The effect of sexual selection on offspring fitness depends on the nature of genetic variation. *Current Biology* **22**, 204–208 (2012).

92. MacLellan, K., Kwan, L., Whitlock, M. C. & Rundle, H. D. Dietary stress does not strengthen selection against single deleterious mutations in drosophila melanogaster. *Heredity* **108**, 203–210 (2012).

93. MacLellan, K., Whitlock, M. C. & Rundle, H. D. Sexual selection against deleterious mutations via variable male search success. *Biology Letters* **5**, 795–797 (2009).

94. Maklakov, A. A., Bonduriansky, R. & Brooks, R. C. Sex differences, sexual selection, and ageing: An experimental evolution approach. *Evolution* **63**, 2491–2503 (2009).

95. Maklakov, A. A. & Fricke, C. Sexual selection did not contribute to the evolution of male lifespan under curtailed age at reproduction in a seed beetle. *Ecological Entomology* **34**, 638–643 (2009).

96. Maklakov, A. A., Fricke, C. & Arnqvist, G. Sexual selection affects lifespan and aging in the seed beetle. *Aging Cell* **6**, 739–744 (2007).

97. Mallet, M. A., Bouchard, J. M., Kimber, C. M. & Chippindale, A. K. Experimental mutation-accumulation on the x chromosome of drosophila melanogaster reveals stronger selection on males than females. *Bmc*

Evolutionary Biology **11**, (2011).

98. Mallet, M. A. & Chippindale, A. K. Inbreeding reveals stronger net selection on drosophila melanogaster males: Implications for mutation load and the fitness of sexual females. *Heredity* **106**, 994–1002 (2011).

99. Martin, O. Y. & Hosken, D. J. Reproductive consequences of population divergence through sexual conflict. *Current Biology* **14**, 906–910 (2004).

100. Matsuyama, T. & Kuba, H. Mating time and call frequency of males between mass-reared and wild strains of melon fly, bactrocera cucurbitae (coquillett) (diptera: Tephritidae). *Applied Entomology and Zoology* **44**, 309–314 (2009).

101. McLain, D. K. Population density and the intensity of sexual selection on body length in spatially or temporally restricted natural populations of a seed bug. *Behavioral Ecology and Sociobiology* **30**, 347–356 (1992).

102. Meffert, L. M., Regan, J. L., Hicks, S. K., Mukana, N. & Day, S. B. Testing alternative methods for purging genetic load using the housefly (musca domestica l.). *Genetica* **128**, 419–427 (2006).

103. Michalczyk, L. *et al.* Inbreeding promotes female promiscuity. *Science* **333**, 1739–1742 (2011).

104. Morrow, E. H., Stewart, A. D. & Rice, W. R. Assessing the extent of genome-wide intralocus sexual conflict via experimentally enforced gender-limited selection. *Journal of Evolutionary Biology* **21**, 1046–1054 (2008).

105. Nandy, B. *et al.* Experimental evolution of female traits under different levels of intersexual conflict in drosophila melanogaster. *Evolution* **68**, 412–425 (2014).

106. Nie, H. & Kaneshiro, K. Sexual selection and incipient speciation in hawaiian drosophila. *Science Bulletin* **61**, 125–131 (2016).

107. Palopoli, M. F. *et al.* Natural and experimental evolution of sexual conflict within caenorhabditis nematodes. *Bmc Evolutionary Biology* **15**, (2015).

108. Partridge, L. Mate choice increases a component of offspring fitness in fruit-flies. *Nature* **283**, 290–291 (1980).

109. Perry, J. C. *et al.* Experimental evolution under hyper-promiscuity in drosophila melanogaster. *Bmc Evolutionary Biology* **16**, (2016).

110. Pischedda, A. & Chippindale, A. Sex, mutation and fitness: Asymmetric costs and routes to recovery through compensatory evolution. *Journal of Evolutionary Biology* **18**, 1115–1122 (2005).

111. Pischedda, A. & Chippindale, A. K. Intralocus sexual conflict diminishes the benefits of sexual selection. *Plos Biology* **4**, 2099–2103 (2006).

112. Pitnick, S., Brown, W. D. & Miller, G. T. Evolution of female remating behaviour following experimental removal of sexual selection. *Proceedings of the Royal Society B-Biological Sciences* **268**, 557–563 (2001).

113. Pitnick, S., Miller, G. T., Reagan, J. & Holland, B. Males' evolutionary responses to experimental removal of sexual selection. *Proceedings of the Royal Society B-Biological Sciences* **268**, 1071–1080 (2001).

114. Plesnar-Bielak, A. *et al.* No evidence for reproductive isolation through sexual conflict in the bulb mite rhizoglyphus robini. *PLoS ONE* **8**, (2013).

115. Power, D. J. & Holman, L. Polyandrous females found fitter populations. *Journal of Evolutionary Biology* **27**, 1948–1955 (2014).

116. Power, D. J. & Holman, L. Assessing the alignment of sexual and natural selection using radiomutagenized seed beetles. *Journal of Evolutionary Biology* **28**, 1039–1048 (2015).

117. Price, T. A. R., Hurst, G. D. D. & Wedell, N. Polyandry prevents extinction. *Current Biology* **20**,

471–475 (2010).

118. Prokop, Z. M. *et al.* Do males pay for sex? Sex-specific selection coefficients suggest not. *Evolution* **71**, 650–661 (2017).

119. Promislow, D. E. L., Smith, E. A. & Pearse, L. Adult fitness consequences of sexual selection in drosophila melanogaster. *Proceedings of the National Academy of Sciences of the United States of America* **95**, 10687–10692 (1998).

120. Radwan, J. Effectiveness of sexual selection in removing mutations induced with ionizing radiation. *Ecology Letters* **7**, 1149–1154 (2004).

121. Radwan, J., Unrug, J., Snigorska, K. & Gawronska, K. Effectiveness of sexual selection in preventing fitness deterioration in bulb mite populations under relaxed natural selection. *Journal of Evolutionary Biology* **17**, 94–99 (2004).

122. Rundle, H. D., Chenoweth, S. F. & Blows, M. W. The roles of natural and sexual selection during adaptation to a novel environment. *Evolution* **60**, 2218–2225 (2006).

123. Rundle, H. D., Chenoweth, S. F. & Blows, M. W. The diversification of mate preferences by natural and sexual selection. *Journal of Evolutionary Biology* **22**, 1608–1615 (2009).

124. Rundle, H. D., Odeen, A. & Mooers, A. 0. An experimental test for indirect benefits in drosophila melanogaster. *BMC Evolutionary Biology* **7**, (2007).

125. Savic Veselinovic, M., Pavkovic-Lucic, S., Kurbalija Novicic, Z., Jelic, M. & Andelkovic, M. Sexual selection can reduce mutational load in drosophila subobscura. *Genetika-Belgrade* **45**, 537–552 (2013).

126. Seslija, D., Marecko, I. & Tucic, N. Sexual selection and senescence: Do seed beetle males (acanthoscelides obtectus, bruchidae, coleoptera) shape the longevity of their mates? *Journal of Zoological Systematics and Evolutionary Research* **46**, 323–330 (2008).

127. Sharma, M. D., Hunt, J. & Hosken, D. J. Antagonistic responses to natural and sexual selection and the sex-specific evolution of cuticular hydrocarbons in drosophila simulans. *Evolution* **66**, 665–677 (2012).

128. Sharp, N. P. & Agrawal, A. F. Mating density and the strength of sexual selection against deleterious alleles in drosophila melanogaster. *Evolution* **62**, 857–867 (2008).

129. Sharp, N. P. & Agrawal, A. F. Sexual selection and the random union of gametes: Testing for a correlation in fitness between mates in drosophila melanogaster. *American Naturalist* **174**, 613–622 (2009).

130. Simmons, L. W. & Firman, R. C. Experimental evidence for the evolution of the mammalian baculum by sexual selection. *Evolution* **68**, 276–283 (2014).

131. Simmons, L. W. & Garcia-Gonzalez, F. Experimental coevolution of male and female genital morphology. *Nature Communications* **2**, (2011).

132. Simmons, L. W., House, C. M., Hunt, J. & Garcia-Gonzalez, F. Evolutionary response to sexual selection in male genital morphology. *Current Biology* **19**, 1442–1446 (2009).

133. Snook, R. R., Gidaszewski, N. A., Chapman, T. & Simmons, L. W. Sexual selection and the evolution of secondary sexual traits: Sex comb evolution in drosophila. *Journal of Evolutionary Biology* **26**, 912–918 (2013).

134. Whitlock, M. C. & Bourguet, D. Factors affecting the genetic load in drosophila: Synergistic epistasis and correlations among fitness components. *Evolution* **54**, 1654–1660 (2000).
