# Supplementary material for: Meta-analytic evidence that sexual selection improves population fitness
Source: Nat Commun. 2019 May 1;10:2017. doi: 10.1038/s41467-019-10074-7 (PMC6494874; doi:10.1038/s41467-019-10074-7)
Supplement: Supplementary file 3 — Reporting Summary [file 41467_2019_10074_MOESM3_ESM.pdf]

nature research

Corresponding author(s): Justin G Cally

Last updated by author(s): Mar 13, 2019

Reporting Summary

Nature Research wishes to improve the reproducibility of the work that we publish. This form provides structure for consistency and transparency in reporting. For further information on Nature Research policies, see Authors & Referees and the Editorial Policy Checklist.

Statistics

For all statistical analyses, confirm that the following items are present in the figure legend, table legend, main text, or Methods section.

n/a	Confirmed	
☐	☒	The exact sample size (*n*) for each experimental group/condition, given as a discrete number and unit of measurement
☐	☒	A statement on whether measurements were taken from distinct samples or whether the same sample was measured repeatedly
☐	☒	The statistical test(s) used AND whether they are one- or two-sided<br>*Only common tests should be described solely by name; describe more complex techniques in the Methods section.*
☐	☒	A description of all covariates tested
☒	☐	A description of any assumptions or corrections, such as tests of normality and adjustment for multiple comparisons
☐	☒	A full description of the statistical parameters including central tendency (e.g. means) or other basic estimates (e.g. regression coefficient) AND variation (e.g. standard deviation) or associated estimates of uncertainty (e.g. confidence intervals)
☐	☒	For null hypothesis testing, the test statistic (e.g. *F*, *t*, *r*) with confidence intervals, effect sizes, degrees of freedom and *P* value noted<br>*Give P values as exact values whenever suitable.*
☐	☒	For Bayesian analysis, information on the choice of priors and Markov chain Monte Carlo settings
☐	☒	For hierarchical and complex designs, identification of the appropriate level for tests and full reporting of outcomes
☐	☒	Estimates of effect sizes (e.g. Cohen's *d*, Pearson's *r*), indicating how they were calculated

Our web collection on statistics for biologists contains articles on many of the points above.

Software and code

Policy information about availability of computer code

Data collection	The code used to perform this meta-analysis is freely available on Github (https://justincally.github.io/SexualSelection/). Here the code is available through a neatly compiled html, with the R Markdown file found within the corresponding Github repository: (https://github.com/JustinCally/SexualSelection).
Data analysis	The code used to perform this meta-analysis is freely available on Github (https://justincally.github.io/SexualSelection/). Here the code is available through a neatly compiled html, with the R Markdown file found within the corresponding Github repository: (https://github.com/JustinCally/SexualSelection).

For manuscripts utilizing custom algorithms or software that are central to the research but not yet described in published literature, software must be made available to editors/reviewers. We strongly encourage code deposition in a community repository (e.g. GitHub). See the Nature Research guidelines for submitting code & software for further information.

Data

Policy information about availability of data

All manuscripts must include a data availability statement. This statement should provide the following information, where applicable:

- Accession codes, unique identifiers, or web links for publicly available datasets
- A list of figures that have associated raw data
- A description of any restrictions on data availability

All data are freely available on Github (https://github.com/JustinCally/SexualSelection). The repository provides a source data file that was used to create all results, tables and figures.

Field-specific reporting

Please select the one below that is the best fit for your research. If you are not sure, read the appropriate sections before making your selection.

☐ Life sciences ☐ Behavioural & social sciences ☒ Ecological, evolutionary & environmental sciences

For a reference copy of the document with all sections, see nature.com/documents/nr-reporting-summary-flat.pdf

Ecological, evolutionary & environmental sciences study design

All studies must disclose on these points even when the disclosure is negative.

Study description	A systematic review and mixed-effects meta-analysis.
Research sample	The research sample are the effect sizes from studies discovered by our systematic literature search. The effect sizes are sourced from sexual selection experiments on numerous taxa (e.g., Beetles, Mice, Guppies, Flies, Crickets, Mites). Primary Studies are listed in the Supplementary Material with reasons for inclusion and exclusion in Table S2.
Sampling strategy	Systematic literature search following the PRISMA (preferred reporting items for systematic reviews and meta-analyses). The search strategy is documented in the Supplementary Material.
Data collection	Justin G Cally collected the data by reading the manuscripts/associated data and extracting means, sd/se and sample sizes (if present). Otherwise extracting summary statistics (F, t, chi).
Timing and spatial scale	The literature search was conducted on the 9th June 2017. Only studies published and on Scopus/Web of Science by this date are included in the meta-analysis.
Data exclusions	Following PRISMA guidelines, data was not excluded after the study met the inclusion criteria (see Supplementary Material for details).
Reproducibility	Reproducibility was achieved through multiple statistical approaches (Bayesian and REML).
Randomization	No randomization was used in the systematic review and meta-analysis.
Blinding	Blinding was not utilized during the data extraction phase as a single author was undertaking the data extraction.

Did the study involve field work? ☐ Yes ☒ No

Reporting for specific materials, systems and methods

We require information from authors about some types of materials, experimental systems and methods used in many studies. Here, indicate whether each material, system or method listed is relevant to your study. If you are not sure if a list item applies to your research, read the appropriate section before selecting a response.

Materials & experimental systems

n/a	Involved in the study	
☒	☐	Antibodies
☒	☐	Eukaryotic cell lines
☒	☐	Palaeontology
☒	☐	Animals and other organisms
☒	☐	Human research participants
☒	☐	Clinical data

Methods

n/a	Involved in the study	
☒	☐	ChIP-seq
☒	☐	Flow cytometry
☒	☐	MRI-based neuroimaging
